# High Temporal Resolution Land Use Regression Models with POI Characteristics of the PM_2.5_ Distribution in Beijing, China

**DOI:** 10.3390/ijerph18116143

**Published:** 2021-06-07

**Authors:** Yan Zhang, Hongguang Cheng, Di Huang, Chunbao Fu

**Affiliations:** School of Environment, Beijing Normal University, Beijing 100875, China; yanzlcm@163.com (Y.Z.); tinyjasmine@163.com (D.H.); fucb1928@163.com (C.F.)

**Keywords:** particular matter, land use regression, point of interest, temporal resolution, exposure

## Abstract

PM_2.5_ is one of the primary components of air pollutants, and it has wide impacts on human health. Land use regression models have the typical disadvantage of low temporal resolution. In this study, various point of interests (POIs) variables are added to the usual predictive variables of the general land use regression (LUR) model to improve the temporal resolution. Hourly PM_2.5_ concentration data from 35 monitoring stations in Beijing, China, were used. Twelve LUR models were developed for working days and non-working days of the heating season and non-heating season, respectively. The results showed that these models achieved good fitness in winter and summer, and the highest R^2^ of the winter and summer models were 0.951 and 0.628, respectively. Meteorological factors, POIs, and roads factors were the most critical predictive variables in the models. This study also showed that POIs had time characteristics, and different types of POIs showed different explanations ranging from 5.5% to 41.2% of the models on working days or non-working days, respectively. Therefore, this study confirmed that POIs can greatly improve the temporal resolution of LUR models, which is significant for high precision exposure studies.

## 1. Introduction

Air pollution problems are becoming increasingly serious worldwide. The primary pollutants found in cities include sulfur dioxide, ozone, nitrogen oxides, and inhalable and fine particulate matter. Fine particulate matter is an important air pollutant, which is considered the primary environmental risk factor, and it is the fourth leading risk factor for death and disability in China [1]. In particular, PM_2.5_ has the most significant effect. Long-term exposure to PM_2.5_ pollution can have a severe impact on human health. Numerous epidemiological studies have shown that PM_2.5_ is closely related to a variety of cerebrovascular diseases, respiratory diseases, and immune system diseases, as well as long-term or short-term mortality in hospitals [2,3,4,5]. According to a report in 2014, the PM_2.5_ concentration in 90% of Chinese cities had exceeded the standard of 35 μg·m^−3^, and the average number of exceeded days was 246. Therefore, studies that investigate PM_2.5_ are of great significance for local pollution prevention and targeted health prevention measures.

Various modeling methods, such as spatial interpolation [6], the atmospheric dispersion model [7], satellite inversion [8], and deep learning [9,10,11], have been used to simulate the distribution of regional pollutants. However, many methods, such as diffusion models, require a large amount of data difficult to obtain, and it is difficult to achieve high-precision under the condition of a lack of input data resources [12]. The land use regression (LUR) model is a multivariate regression modeling method based on the observation concentration of air pollutants and its surrounding geographical factors. This method is widely used to estimate the concentrations of outdoor air pollutants since it was improved by Briggs et al. [13]. Due to its high accuracy and relatively low investment [14], LUR models have a wide range of applications. Beelen et al. [15] used an LUR model in the ESCAPE (European Study of Cohorts for Air Pollution Effects) project, which explained the variability of annual NO_2_ and NO_x_ concentrations. Ma et al. [16] used multi-scale LUR models to simulate the NO_2_ concentration in Auckland, which performed better than the universal kriging (UK) model and the inverse distance weighting (IDW) and ordinary kriging (OK) models. Yang et al. [17] developed LUR models to predict ultrafine particle concentrations in London, and the results showed that the LUR models had moderate to good performances within these areas. Saucy et al. [18] used warm and cold season LUR models for NO_2_ and PM_2.5_ concentrations in peri-urban areas in South Africa, and it was demonstrated that the models could be successfully applied in local areas. Weissert et al. [19] developed a microscale LUR model for a heavily trafficked road in Auckland, New Zealand. This research represented the expansibility of small-scale variability in pollutant concentrations. In China, LUR modeling has been shown to have methodological advantages and has become increasingly popular in air pollution studies in recent years [20,21,22].

In human exposure and health risk assessment areas, LUR models are often used to simulate exposure concentrations [23]. High temporal resolution pollution data can improve the precision of epidemiological studies and human exposure research. Although LUR is a reasonable and reliable modelling approach, to some degree it has the disadvantages of low temporal resolution and poor spatial portability [16,24]. The most common spatial predictors in LUR models include elevation, population, road network, and land use. Many studies have confirmed that these predictors can explain most of the models and have strong correlations with pollutant concentrations [20,25,26].

City pollution sources and functions vary from region to region due to the different characteristics of urban layouts [27,28]. In particular, various urban functional areas are comprised of various points of interest (POIs) [29]. Studies have confirmed that different functional areas with different types of POIs have differences in air pollution characteristics and attractiveness to populations [30,31,32]. POIs show typical functional features at different times. For example, people tend to congregate in residential areas in the evening and gather in office places for working during the daytime. Therefore, this may result in different POI predictors related to pollutants at different times. That means that if POI predictors are added into LUR models, this may improve the precision and temporal–spatial resolution. Nori-Sarma et al. [26] used LUR models to predict urban NO_2_ exposure in Mysore, India. The study showed industrial sites and religious POIs, and these high human activity POIs were associated with higher levels of NO_2_. Lu et al. [33] developed three sets of LUR models and the independent variables were common land use data, local business permit data, and Google POI data. The results showed that models that used the Google POI data performed the best.

Beijing is one of the cities with the worst air pollution measures in northern China. Although Beijing has made phased progress in reducing the air pollution problem in recent years, it still exceeds the minimum limit of the China national standard (GB 3095-2012). Unlike previous studies that have used average annual and seasonal pollutant concentrations, this study uses hourly pollution concentrations from the heating season, the non-heating season, working days, and non-working days. In these LUR models, in addition to conventional predictors, such as land use, meteorological factors, population and elevation, POIs are also included. This study aims to improve the temporal resolution and model accuracy and simulate the PM_2.5_ distribution in Beijing during different time circumstances, and to provide guidance and reference value for continued LUR and exposure studies.

## 2. Materials and Methods

### 2.1. Study Area

The Beijing, Tianjin, and Hebei regions are the most polluted areas in northern China. Beijing is the capital of China, and it has representativeness and data availability. It has 16 districts with a total area of 16,410.54 square kilometers at a latitude of 39°26′–41°03′ and longitude of E115°25′–117°30′. The permanent resident population is 21.536 million, and the urban population is 18.65 million, accounting for 86.6 percent of the total urban population. The terrain of Beijing primarily consists of plains and mountainous areas, and is high in the northwest and low in the southeast. Beijing is surrounded on three sides by mountains to the west, north, and northeast, and to the southeast by a plain that slopes gently toward the Bohai Sea. The terrain conditions make it easy for various pollutants to accumulate and difficult to diffuse. Beijing has a warm temperate semi-humid and semi-arid monsoon climate, with high temperatures and rain in summer and cold and dry weather in winter.

### 2.2. PM_2.5_ Data

**Figure 1:**
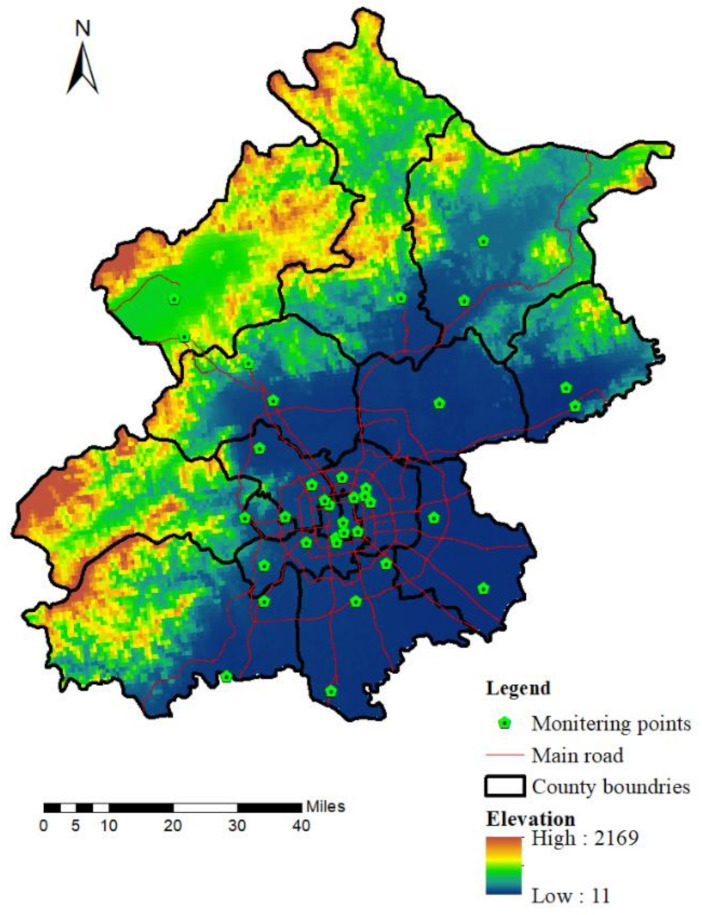
Study area and distribution of the monitoring stations.

Beijing has 35 air quality monitoring stations. There are 24 environmental assessment and control stations, six regional stations, and five traffic monitoring stations. Environmental assessment stations represent the overall level and variations in the regional environment. Six regional stations represent the level of regional concentrations, and the five traffic monitoring stations were used to measure the influence of road traffic pollution sources on the ambient air quality distributed in different ring lines and primary roads. The locations of the monitoring stations are shown in Figure 1. The monitoring stations were primarily concentrated in the central urban area and were distributed evenly in the suburbs. The hourly PM_2.5_ concentration data came from the Beijing Municipal Environmental Protection and Testing Center (http://www.bjmemc.com.cn (accessed on 10 December 2019)).

Hourly pollutants concentration was used to develop LUR models. Because of its high temporal resolution, PM_2.5_ distribution showed huge differences due to season and time characteristics. Considering high time scale POI influence and corresponding to meteorological condition variation, this study chose working days and non-working days from winter heating season and summer non-heating season which were randomly chosen at 0:00, 8:00, and 18:00. These corresponded to midnight, morning rush, and evening rush. The average PM_2.5_ concentration at these three times on 3 August 2019 represented a summer non-working day. 6 August 2019 represented a summer working day. In the same way, 12 and 14 January 2019 represented a winter non-working day and working day, respectively. Missing values from some stations at a particular moment were removed. Given the above, 12 LUR models in total were developed.

### 2.3. Predictors and Data Source

Source apportionment studies have shown that PM_2.5_ pollutants are primarily caused by various sources, including traffic, coal combustion, biomass burning, industrial sources, and dust [34,35]. In addition, meteorological conditions, local emissions, regional transport, and complex topography can also affect the formation of pollutants [36,37]. This study summarized the common geographic variables used in previous studies. Buffer sizes with radii of 300 m, 500 m, 600 m, 700 m, 800 m, 900 m, 1000 m, 1300 m, 1500 m, 2000 m, and 2500 m centered from the monitoring station were established. The predictors included land use area, road length, number of different POIs, industrial pollution sources, meteorological factors, elevation, and population. Specific details of these predictors can be seen in Table 1.

Land use data was obtained from the Chinese resource and environment data cloud platform (http://www.resdc.cn/ (accessed on 10 December 2019)). Beijing primarily contains the following six land types: arable land, garden land, woodland, grassland, commercial service land, industrial and mining warehousing land, and residential land. Meteorological data, including temperature, relative humidity, air pressure, and wind speed, came from the China meteorological science data center (http://data.cma.cn/en (accessed on 10 December 2019)). Meteorological data from 33 stations in Beijing (15 stations) and its surrounding areas (18 stations in Hebei and Tianjin) were utilized. The Kriging interpolation method was used to simulate the annual mean values of the 35 monitoring stations. The road network data came from the National Geomatic Center of China (http://www.ngcc.cn/ngcc/ (accessed on 10 December 2019)). These data contained the name, type, length, and other information on all the roads in Beijing. While vehicle flow information was not available, the length of roads within the buffer zone of the monitoring station is a good substitute [17,38]. By considering traffic flow and geographical location information, the roads were divided into two categories. Expressways, national roads, provincial roads, county roads, and urban expressways were merged into primary roads, township roads, and pedestrian roads, and the other roads were merged into the secondary roads. The length of the various roads in each buffer zone were obtained as road factors using the GIS spatial superposition method. The POI data was obtained using an application called the Baidu map using Python. There were 13 types of POI, including catering services, scenic spots, public facilities, companies, shopping places, transportation, financial banks, science and education places, commercial and residential housing, life services, sports and leisure, medical care, and government agencies. The Shuttle Radar Topography Mission (SRTM) digital elevation data (90 m spatial resolution) was used as digital elevation model (DEM). The population density data was based on the population distribution dataset of a 1 km grid in 2015. The industrial pollution sources data was obtained from the website of the Ministry of Environmental Protection. This contained basic information on the national key monitoring enterprises of waste gas.

### 2.4. Land Use Regression Modelling

The land use regression (LUR) model is based on the least square method to establish a regression equation between air pollutants at monitoring points and the characteristic predictors of the surrounding environment near these points. Then, by using the characteristic predictors of the surrounding environment at unknown points, the model algorithm of pollutant concentrations at unknown points can be estimated by the equation. The model equation is as follows:

*y* = *β*_0_ + *β*_1×1_ + *β*_2×2_ + … + *β*_n_*x*_n_ + *α*, (1)

where *y* represents the PM_2.5_ concentration value; *x*_1_, *x*_2_, …, *x*_n_ represents the influence factors in the model; and *α* is a random variable. First, a correlation analysis was conducted between the PM_2.5_ data and corresponding predictors of the station. In this study, the Spearman correlation coefficient was used as the standard to select these predictors. To reduce the possibility of collinearity between variables belonging to the same category, the predictor with the highest correlation coefficient in each category of the buffers was kept, and the rest were eliminated [39,40]. A stepwise linear regression was performed on the screened predictors to obtain the multiple linear regression equations. The significance level (*p*-value < 0.05) and variance inflation factor (VIF < 5) of each predictor that remained in the models were used to check their significance levels and ensure no issues of multicollinearity. Finally, the models’ fitness degrees were judged using R^2^, and the root-mean-square error (RMSE) was used to evaluate the prediction accuracy of the models. In addition, the partial R^2^ of each predictor was used to judge the overall explanation of the model by this factor.

### 2.5. Validation and Evaluation

This study used the 10-fold cross validation method. The principle of the 10-fold cross validation is to divide the entire data set into 10 parts, of which nine were selected as the training set and one as the prediction set. The RMSE and the adjusted R^2^ were used to evaluate the predictive accuracy and fitness of the models through many times of repeat training. R software was used to conduct all of the statistical analyses.

When the final LUR model was obtained, the regression equation was used to simulate the concentration of pollutants at non-monitored points. This process is called regression mapping [41]. This method can better simulate the spatial distribution of air pollutants. A grid of 3 km and 3 km within the research area was generated, and the corresponding impact factor values of each grid point were obtained. These were then placed into the obtained multiple regression equation to predict the PM_2.5_ concentration. Then, the Kriging interpolation method was used to simulate and generate the spatial distribution of the predicted PM_2.5_ concentration at different times during working days and non-working days in winter and summer in the study area.

## 3. Results

### 3.1. Descriptive Statistics for the PM_2.5_ Data

Table 2 shows the basic descriptive statistics of the PM_2.5_ at different times during winter working days, non-working days, summer working days, and non-working days. The PM_2.5_ pollutant data of each district at different times can be seen in Table S1. As a whole, there was a large difference in the study area in the PM_2.5_ concentrations between winter and summer. According to China’s GB 3095-2012, it can be seen that the pollution concentration during the two winter days seriously exceeded the standard, and the average PM_2.5_ value ranged from 95.9 to 323.4 μg·m^−3^. The median values of the six moments in the two winter days were generally high, the upper and lower quartile intervals had a larger span, and the PM_2.5_ concentration values of the different monitoring sites had a greater degree of dispersion. In comparison, the PM_2.5_ concentration did not exceed the standard at six times on two summer days. The average PM_2.5_ value was between 22.4–46.6 μg·m^−3^, the maximum was between 42–69 μg·m^−3^, and the minimum was between 9–25 μg·m^−3^. In general, the PM_2.5_ concentration at the different monitoring stations in the study area was discrete.

After a normality test of the several sets of data, a Student’s *t*-test and Mann–Whitney U test were performed on the PM_2.5_ concentration at three times of the day according to the normal distribution of the PM_2.5_ concentration. During winter, there was no significant difference between 0:00 and 18:00 on winter workdays (*p* > 0.05). In addition, there were significant differences between 0:00 and 8:00 and between 8:00 and 18:00 (*p* < 0.05). There were significant differences in the PM_2.5_ concentration of the three non-working days in winter (*p* < 0.01). It can be seen that in winter, whether it was a working day or a non-working day, the PM_2.5_ concentration at the three times all presented a trend, with the strongest at 18:00 and the lowest concentration at 8:00. There was no significant difference between 0:00 and 18:00 in summer working days and non-working days (*p* > 0.05), but there were significant differences between 0:00 and 8:00 and 8:00 and 18:00 (*p* < 0.05). This meant that at different times in one day there were large differences in the PM_2.5_ concentrations at the same station.

### 3.2. LUR Models

Correlation analysis results can be seen in Table S2. Table 3 shows the results of a total of 12 LUR models at three times during winter working days, non-working days, summer working days, and non-working days. Overall, the fitness of the LUR models at various moments in winter were better than in summer, with the adjusted R^2^ ranging from 0.644 to 0.951, and the 10-fold cross-validation adjusted R^2^ ranging from 0.671 to 0.947. The adjusted R^2^ of the summer time models were between 0.312 and 0.628, and the 10-fold cross-validation adjusted R^2^ were between 0.299 and 0.605. In summary, the R^2^ varied greatly at different times, even in the same season. Compared with the summer LUR models, the RMSE of the winter models were generally larger. The winter models’ RMSE ranged from 19.79 to 50.76 μg·m^−3^, and the 10-fold cv RMSE were between 14.52 to 55.42 μg·m^−3^. This was related to winter’s higher concentration of pollutants in the study area. In the summer models, the RMSE were between 5.08–8.24 μg·m^−3^, and the 10-fold cv RMSE were between 4.84–9.55 μg·m^−3^. For the working days and non-working days during the same season, the R^2^ and RMSE gaps were not significant.

Table 4, Table 5, Table 6 and Table 7 show the final predictors of 12 LUR models. The final predictors retained in the LUR models included meteorological factors, roads, land use types, POIs, and elevation according to the correlation analysis. The partial R^2^ represented the contribution of the predictive variables in each model to the model. The meteorological factors were included in nearly all the models. During winter, the LUR models at different times and relative humidities were included in all six models, and this explained 54–86% of the variations. The second meteorological factor was temperature. Unlike the winter models, the meteorological factors had little effect on the summer LUR models, although the relative humidity and temperature were both included. This may have been due to the different dominant factors of the PM_2.5_ concentration in the different seasons. At 0:00 on the summer working days, the temperature explained 19.3% of the variations in the model. However, at 0:00 and 8:00 on non-working days in summer, the temperature and relative humidity only explained 6.9% and 6.3%, respectively, of the variations in the models. Land use and road data also explained a large portion of the model results. Grassland was included in the winter and summer models, accounting for 5.6% and 6.3% of the variations in the models, respectively, both of which were negatively correlated with the PM_2.5_ concentrations. Road data were included in both the winter and summer models; at 8:00 a.m. on winter and summer working days, and at 18:00 on summer non-working day. The models that included road data were both in the morning and evening peaks and explained 3.5%, 15.6%, and 8.3% of the variations in the models.

The POIs were included in the models of both seasons and had a certain influence on the PM_2.5_ concentrations. Eleven types of POIs were included: residential housing, catering services, life services, scenic spots, medical care, public facilities, science and education places, sports and leisure, shopping places, government agencies, and financial banks. The results showed that on working days or non-working days in the different seasons, different POIs had a certain influence on the PM_2.5_ concentration, and their relationships to the pollutants at specific moments had diversity. Compared with the summer results, the number of POIs included in the winter models was smaller because the relative humidity contributed most to the winter models. At 0:00 on a winter working day, sports leisure within 2500 m explained 7.3% of the variation in the model. At 0:00 on non-working days in winter, such POIs explained 20.1% of the variation in the model. At 8:00 and 18:00 on non-working days in winter, catering services and shopping places were included in the models, which explained 5.5% and 6.8%, respectively, of the variation. Furthermore, POIs were included in all the summer models, and these explained a great deal of the variations. At 0:00 on summer working days and non-working days, commercial residences explained 41.2% and 39.8% of variation in the model, respectively. This was consistent with the feature that most people gather in residential areas at midnight. At 8:00 on a summer working day, financial banks within 3000 m, government agencies within 300 m and life service within 300 m explained 8.2%, 6.5%, and 5.9% of the variation in the model, respectively. At 18:00 on a summer working day, all of the contributions to the PM_2.5_ pollutants came from the POIs, including commercial residences and scenic spots, explaining 25.7% and 9.7% of the variation in the model, respectively. At 8:00 on a non-working day in summer, the POIs were still important sources of PM_2.5_ pollutants. Medical care and public facilities explained 45.6% and 11.6% of the variation in the model, respectively. At 18:00 on summer working days, science and education places explained 51% of the LUR model. In summary, POIs have greatly improved the time resolution of LUR models for different moments.

### 3.3. PM_2.5_ Evaluation and Distribution

**Figure 2:**
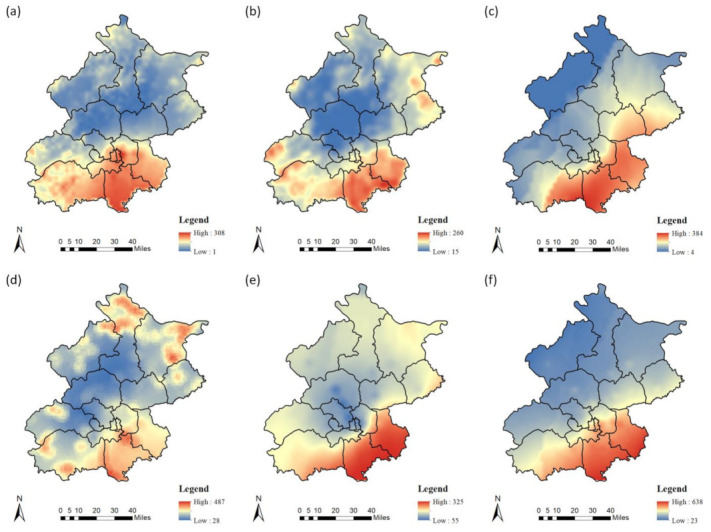

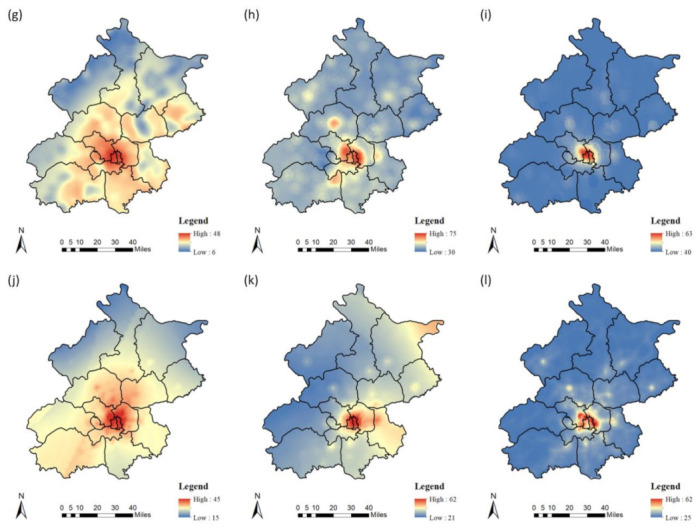
Spatial distribution of PM_2.5_ during the different seasons and times. (**a**–**c**) show the spatial distribution of PM_2.5_ during winter working days at 0:00, 8:00, and 18:00; (**d**–**f**) show the spatial distribution of PM_2.5_ during winter non-working days at 0:00, 8:00, and 18:00; (**g**–**i**) shows the spatial distribution of PM_2.5_ during summer working days at 0:00, 8:00, and 18:00; and (**j**–**l**) shows the spatial distribution of PM_2.5_ during summer non-working days at 0:00, 8:00, and 18:00.

The center value of each grid point in the study area was estimated according to the regression equation of the LUR models. The Kriging interpolation method was used to predict the spatial distribution of PM_2.5_ in the study area. Figure 2 shows the temporal and spatial distribution of the PM_2.5_ at different times during winter working days and non-working days in Beijing. It can be seen that distribution of PM_2.5_ in study area had obvious temporal and spatial heterogeneity. Even in the different seasons, the pollutants were higher in the southeast and lower in the central urban and northwest mountainous areas. This was consistent with the high terrain in the northwest and gentle terrain southeast of the study area. This feature was more significant in winter. In summer, the high PM_2.5_ concentration is primarily concentrated in urban areas and south of the city, but it was more obvious in urban centers.

**Figure 3:**
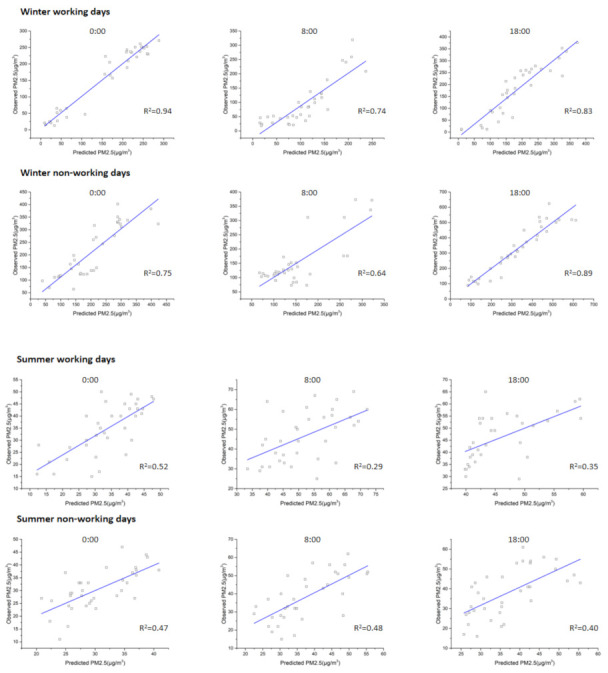
Scatter plots of the PM_2.5_ predictions from the Kriging method and the monitoring observations.

Figure 3 shows the Kriging’s predicted PM_2.5_ data compared with the actual values. The scatter plots of the predicted versus observed PM_2.5_ data are displayed. Although there were some outliers, as a whole it can be seen that the explanatory ability of the Kriging expansion was basically the same or slightly improved compared to that of the LUR models, according to a comparison of the PM_2.5_ concentration predicted values with actual values by Kriging interpolation. By using the linear regression prediction of the PM_2.5_ concentration at the different moments, the prediction values of 0:00, 8:00, and 18:00 on winter working days ranged from 9.27–288.29 μg·m^−3^, 15–236.51 μg·m^−3^, and 10–373.33 μg·m^−3^ respectively, with mean values of 160.11 μg·m^−3^, 106.98 μg·m^−3^, and 180.02 μg·m^−3^, respectively. The relative error between the forecast mean value and the actual value was −0.94%, 11.63%, and 1.66%, respectively. The prediction value at 0:00, 8:00, and 18:00 during the winter non-working days ranged from 39.46–423.12 μg·m^−3^, 67.60–322.18 μg·m^−3^, and 85.89–612.17 μg·m^−3^ respectively, with mean values of 205.84 μg·m^−3^, 150.53 μg·m^−3^, and 323.52 μg·m^−3^, respectively. The relative error between the predicted means and actual values were −2.77%, 0.35%, and 0.06%, respectively. In the winter models, the monitoring stations for the predicted maximum and minimum PM_2.5_ values were consistent with the monitoring stations for the actual maximum and minimum values. However, there were more outliers in summer working days and non-working days which illustrated summer models ‘predictive effect. The prediction effect during summer time was worse than the original LUR models. The prediction ranges of the PM_2.5_ concentration at 0:00, 8:00, and 18:00 during the summer working days were 12.22–47.91 μg·m^−3^, 33.46–72.21 μg·m^−3^, and 39.87–59.56 μg·m^−3^, with mean values being 33.36 μg·m^−3^, 52.04 μg·m^−3^, and 45.98 μg·m^−3^, and relative prediction errors of −3.26%, 11.68%, and 0.36%, respectively. The predicted range of the PM_2.5_ concentration at 0:00, 8:00, and 18:00 on the non-working days during summer were 20.87–40.87 μg·m^−3^, 22.50–55.43 μg·m^−3^, and 25.73–55.47 μg·m^−3^, with mean values of 30.36 μg·m^−3^, 37.31 μg·m^−3^, and 37.01 μg·m^−3^, respectively. The relative errors with the actual values were −0.03%, −1.82%, and −2.82%, respectively. In summary, the prediction accuracy of the summer models was relatively high, but the predicted maximum and minimum PM_2.5_ stations were not completely consistent with the actual stations due to the small difference in the overall PM_2.5_ concentration distribution during summer in the study area.

## 4. Discussion

The LUR models in this study well explained the temporal and spatial variations in the PM_2.5_ pollution in the study area, and hourly time precision was achieved. The winter models fitted best, and the explanatory degree of the models at several different moments ranged from 67.5% to 95.5%. These models were highly comparable compared with the LUR models utilized in previous studies. The explanation of these models was higher than the 53% explanation of the annual PM_2.5_ LUR simulation for the United States [42] and higher than 11.4–46.5% of the seasonal average PM_2.5_ models for Bangkok, Thailand [43]. In China, the model explanations were higher than that of Cai et al. [44] to explain 65% of the spatial variability in the PM_2.5_ in Taizhou, China and higher than the explanation of 61% of the PM_2.5_ of the Liaoning central urban agglomeration in the model of Shi et al. [20]. This difference can typically be explained by the measuring concentrations, predictive variables, and original variability of the geographical and socio-economic characteristics of the study area [45].

The models for the two seasons were quite different. PM_2.5_ pollutants in Beijing primarily originated from man-made emissions, including coal combustion, gasoline and diesel vehicle emissions, secondary source pollution, and straw burning on farmlands. Winter is heating season in Beijing. Due to coal combustion from Beijing and its surrounding areas, as well as less vegetation than in summer [46], the concentration of PM_2.5_ in winter was significantly higher than in summer. In addition, Beijing is affected by the high pressure from Mongolia during winter, which leads to the local accumulation of pollutants. During summer, the source of fine particulate matter primarily originates from regional traffic sources, of which there is also a portion from cross-regional transportation. Lv et al. [47] conducted a high-time resolution particle source apportionment in Beijing showed that Baoding and Langfang in Hebei Province contributed significantly to the short-distance transportation, and this portion was not included in these models. This demonstrated that the explanation of several summer models in this study ranged between 35.4% and 66.8%, which was worse than that in winter. These results were consistent with the seasonal models of Shi et al. [20] in which their LUR models explained 61% of the variability in winter and 52% of the variability in summer. The cross-regional transport of traffic pollution sources also explained why the models at 0:00 were greater than 8:00 on the same working days in this study. This was because during the morning rush hour at 8:00, many people from the surrounding areas enter Beijing for work, and the traffic influence produced by this portion could not be included in the models. However, the PM_2.5_ pollutants at 0:00 were primarily from local sources, and nearly each impact factor was included in the model.

Previous studies have generally used the RMSE to determine the accuracy of the models [45], but for LUR models with different spatial and temporal scales, a direct comparison of the RMSE values may have great variability. In this study, the RMSE in the winter models was generally larger than that in summer, which was due to the different background values of the pollutants in the different seasons and the spatial differences of the pollutants. Since the terrain in Beijing is high in the northwest and low in the southeast, the different topography and underlying surface affect the transmission of air pollutants [48]. In the suburbs, human activities are less affected, and the high concentration areas are primarily in the main urban area and the southern plain. As a result, the PM_2.5_ concentration fitted by this model in winter reached the highest level of nearly 700 μg·m^−3^, while the minimum area was less than 10 μg·m^−3^. The RMSE of our summer models ranged from 5.08 μg·m^−3^ to 8.24 μg·m^−3^, which was not significantly different from previous studies in China [20], but was larger than the figures from other global research [45]. This was because the background level of PM_2.5_ in China is several times or even dozens of times higher than that in Europe and the United States. The RMSE of the winter model was also larger than summer due to above reasons.

The predictive variables in the different LUR models are typically not constant due to city-specific conditions and the availability of data. Generally, most LUR studies have used annual or seasonal values. One reason is that the non-contemporaneous measurements of monitoring stations may cause temporal variability [44]. Therefore, one significant drawback of LUR models is their low temporal resolution. The simplest method to calibrate pollutant concentrations are using observations from fixed continuous monitoring stations [49]. This combined with meteorological, satellite data or adding other time-dependent predictive variables [50,51] can also improve the temporal resolution. Since the POIs presented different functional characteristics at different times, this study assumed that different types of POI variables also have time characteristics, and hourly LUR models were developed. The results showed that POIs explained the variation in pollutant concentrations at different times. Before modeling, the 13 types of POIs included in this study were all correlated with PM_2.5_ concentration, and 11 types of POIs were finally included in the models. The association between POIs and pollutants and its temporal characteristics are better reflected by the attractions of POIs to the crowd [29], which will affect the behavior of the crowd, thus explaining the variability of air pollution at that moment. The POIs included in the models, such as shopping, entertainment, medical care, science, education and culture, all have a large traffic flows near these places in daily life. The pollution produced by these vehicle traffic sources contributes to the PM_2.5_ pollution at this moment to a large extent.

In terms of the time characteristics of POIs, it can be seen from the results that at 0:00 on summer working days and non-working days, the commercial and residential housing variables were included. This was due to the poor mobility of people in the urban area at this moment, and the crowd being concentrated near the residences. Hence, there will be some traffic source emissions. However, commercial residential areas contain a lot of commercial office buildings. In many high-tech industrial companies in Beijing, it is a normal phenomenon to work and commute after 0:00. For example, in areas such as Xierqi and Houchangcun in the Haidian District, the taxi rush often occurs after 0:00, and this is also a particular social phenomenon in China. It was also confirmed that on weekdays, commercial residence explained 41.2% of the PM_2.5_ at that time, while on non-weekdays, the explanation was 39.8%. At 18:00 on summer working days, the commercial and residential variable explained 25.7% of the model. This was because people got off work during evening rush hour, and the traffic flow around business buildings and residential areas increased. In addition, there were many buses stops nearby, and this also caused pollutant increases. At 0:00 on working days and non-working days in winter, the POIs explained little of the model ranging from 5.8% to 14.3%. This was primarily because the higher background concentration of pollutants during winter is several times higher than the same time in summer. Compared with meteorological conditions, the POIs contributed a smaller proportion to the PM_2.5_. Catering service POIs were also included in these models. Previous LUR models for Vancouver, Canada, and Europe have also considered the number of restaurants near the monitoring sites [38,52]. Like automobile exhaust, cooking fumes are also a major source of air pollution [53]. The emission inventory performed by Jin et al. [54] showed that in 2017, the catering industry in China released approximately 38.2 kt PM_2.5_ and 47.8 kt PM_10_. In this study, at 8:00 on non-working days in winter, the catering POIs explained 5.5% of the model. On non-weekdays, the passenger flow of various restaurants was more than weekdays, which also increased the emissions of restaurant lampblack. At other times during the non-working day, such as in shopping places, medical care and public facilities were also included in the model. This was because on holidays, people tend to go to these places, and many people will drive private cars or take a taxi, which cause large increases in traffic flow near these POIs and this explains the contribution of the above POIs to the entire model. For example, at 8:00 on non-working days in summer, medical care POIs explained 45.6% of the model, and public facilities POIs explained 11.6%. However, there were several types of POIs included in the model that had no obvious correlation with the places where people gather at that time. For example, at 18:00 on non-working days in summer, there should be few people in schools, but science and education POIs at that time explained 51% of the model. This is because Beijing’s super-large city characteristics lead to POI overlaps in the region. Many schools and educational institutions in Beijing are primarily located in urban areas. These POIs are not independent, and there will be other functional areas nearby, which make the pollutant source unclear. In conclusion, this study confirmed that POIs had a temporal attribute because various POIs show different attractions to the crowd.

Meteorological variables also had time attributes in the models. Meteorological conditions are the primary factors that affect the variability of pollutants with high time resolution. Studies have shown that meteorological conditions contribute greater than 70% of the daily average concentration of pollutants in China [55]. In this study, nearly every model included meteorological variables. The relative humidity was included in all the winter models, and temperature and relative humidity were included in the summer models. Generally, except in the case of precipitation, the greater the relative humidity, the more particles attach to the water vapor, which increases the mass concentration. In the winter models, the relative humidity explained the PM_2.5_ concentration between 54.9% and 86.7%, and all of them were positively correlated. The influence of air temperature on pollutants is complex and often plays a role in combination with wind speed, terrain, atmospheric junction, and other factors. The results of this model showed that temperature at 0:00 in summer was positively correlated with PM_2.5_ concentration, which may have been due to air cooling at night, but land surface temperatures are higher, which makes it difficult for pollutants to diffuse. Statheropoulos et al. [56] analyzed the air pollution factors in Athens, and the results showed that the pollutants had a significant relationship with relative humidity and wind speed. However, in many annual LUR models, meteorological variables are not included [44,57]. This may be due to the large temporal and spatial variability of meteorological conditions, and the impact on pollutants is more reflected at daily or hourly concentrations.

Road data also had a temporal attribute in these models. Theoretically, traffic load and vehicle density data may be significant indicators that reflect vehicle exhaust emissions during a short period of time. However, due to their complexity and unavailability, road lengths were used to represent the traffic emissions. Actually, this method has achieved good results in many studies [17,38,58]. In this study, the road variables were divided into primary roads and secondary roads according to the width and vehicle capacity, and these two types of roads were all included in the models. The models containing this variable were all at 8:00 and 18:00, when the traffic flow was in the peak period and a large number of vehicle exhaust emissions become an important source of air pollutants. As can be seen from the results, the included variable buffers of the primary road were relatively large, 1500 m and 2500 m, while the buffer of the second road was only 300 m. The differences in the buffer radius reflected the characteristics of primary and secondary pollutant emissions. For the PM_2.5_, both primary and secondary emissions can significantly increase its concentration [59]. Different road types were reflected using their functions. The primary roads included urban expressways, national highways, and provincial highways, and they are mainly busy suburban roads. Due to various and complex types of motor vehicle emissions and high traffic densities, large buffer radii may be the result of the secondary discharge of pollutants. However, the secondary roads primarily included a wide distribution of roads, with a small flow of motor vehicles that resulted in a smaller possibility of pollutants spreading in a large area [60]. Thus, they tended to affect the monitoring data in a small area.

Different from previous LUR studies, land, population, and elevation had little influence on the models in this study. This was because these models were based on the hourly time scale of PM_2.5_ concentration, while predictors such as land use and population were more represented in annual or seasonal models. Son et al. [61] improved the LUR models on an hourly time scale in Mexico, which primarily included temporal variables, such as hourly traffic density, meteorological, and holiday variables. Factors such as land use and population had no significant effect. This is consistent with the results of this study.

There currently exist few LUR studies based on hourly pollutant concentrations. In the short-term or more precise individual exposure studies, using annual or seasonal models may lead to deviations [62]. The biggest breakthrough of this research was the realization of the hourly temporal LUR models, which provided a more accurate method for short-term exposure studies and more accurate micro-environment individual exposure studies. High-resolution simulation of regional pollutant concentrations is of great significance for travel prevention and control of residents. Currently in China, there are hourly concentration limits for pollutant emissions. In the future, there may be more precise timescale upgrades to healthy concentration standards. In addition, more pollution emission sources were identified within a short period of time, and this proved the temporal characteristics of the POIs in the models, which has significance for more accurate pollution prevention and control measures. Though we carried out LUR models for PM_2.5_ and there still many researches focused on multiple pollutants, this study provides a new methodological perspective for other types of high-resolution pollutant models which is the most important significance.

However, this study still has some limitations. The best explanation of variability in the summer models was only 66.8%. Hence, there are other factors that remain to be explored during a short period of time. Second, only 35 monitoring data were used in this study, and some data were missing. Hoek et al. [45] recommended using 40–80 pollutant monitoring stations. Additional modeling was performed at other times, but not each model could be successfully applied due to the models’ high time resolution and large pollutants variability. More accurate time variables are required to be explored to optimize the LUR models. Finally, due to the progressive linear regression principle of the LUR model itself, the model assumes a linear relationship between all predictive variables and pollutants, which is inherently limited for some variables. All of these problems require further study.

## 5. Conclusions

In this study, 12 high-resolution LUR models based on the hourly PM_2.5_ concentration were developed. The results showed that, even within the same day and at different times in different seasons, the PM_2.5_ concentration at the same monitoring station varied greatly. The fitness of the winter models at various times were better than that in summer, and the model explanation varied between 67.5% and 95.5%. The summer models explained 35.4–66.8% of the variability. The model results of the different seasons and times were quite different, and the model fitness were not robust. The RMSE of the winter models ranged from 19.79 μg·m^−3^ to 50.76 μg·m^−3^, while the RMSE of summer models ranged from 5.08 μg·m^−3^ to 8.24 μg·m^−3^. The final predictive variables showed that there were differences in explanations of the PM_2.5_ during the different seasons, and the explanations of the meteorological factors to the winter models were the most significant, ranging from 54% to 86%. The road length and POIs also contributed to the winter models, but the explanations were not significant due to winter’s high background value. The summer models showed that the POIs and road length were the primary contributors to the PM_2.5_ pollutants. Pollutant’s transportation is also an important reason. POIs have temporal characteristics, and the contribution of different POIs to the air PM_2.5_ is different on working days or non-working days. In these models, the POIs explained 5.5–51.0% of the variability. This study confirmed that POIs can improve the temporal resolution of LUR models, and this is significant for short-term exposure studies and microenvironment individual exposure studies.

## Figures and Tables

**Table 1:** Predictors in the LUR models.

Category	Predictor Variable	Unit	Buffer Radius
Land use	Arable land	m^2^	300 m, 500 m, 600 m, 700 m, 800 m, 900 m, 1000 m, 1300 m, 1500 m, 2000 m, 2500 m
	Garden land	m^2^	
	Woodland	m^2^	
	Grassland	m^2^	
	Commercial service land	m^2^	
	Industrial and mining warehousing land	m^2^	
Road	Primary road	m	
	Secondary road	m	
POIs	Catering services	/	
	Scenic spots	/	
	Public facilities	/	
	Companies	/	
	Shopping places	/	
	Transportation	/	
	Financial banks	/	
	Science and education places	/	
	Commercial and residential housing	/	
	Life services	/	
	Sports and leisure	/	
	Medical care	/	
	Government agencies	/	
Meteorological factors	Temperature	°C	/
	Relative humidity	%	
	Air pressure	hPa	
	Wind speed	m·s^−1^	
Population	Population	/	
Elevation	Elevation	m	
Industry source	Industry source	/	

LUR: Land use regression; POIs: Point of interests.

**Table 2:** Summary of the descriptive statistics of the PM_2.5_ data in the study area.

Day	Time	Mean ± SD	Max	Min	Median	IQR
Winter working days	0:00	159.5 ± 95.6	271.0	13.0	207.0	53.8–237.8
	8:00	95.9 ± 78.9	319.0	18.0	66.0	43.8–127.5
	18:00	177.5 ± 107.8	376.0	10.0	184.0	84.0–258.0
Winter non-working days	0:00	213.5 ± 103.2	402.0	64.0	171.0	123.3–321.5
	8:00	151.1 ± 84.7	373.0	73.0	118.0	105.5–152.0
	18:00	323.4 ± 159.3	624.0	88.0	313.5	157.8–466.5
Summer working days	0:00	34.5 ± 10.6	50.0	15.0	35.0	27.5–43.0
	8:00	46.6 ± 12.7	69.0	25.0	45.0	34.5–56.5
	18:00	38.5 ± 7.5	49.0	9.0	40.0	37.5–42.5
Summer non-working days	0:00	30.4 ± 7.8	47.0	11.0	29.0	26.0–36.5
	8:00	38.0 ± 12.9	62.0	15.0	37.0	28.0–49.8
	18:00	22.4 ± 7.5	42.0	10.0	23.0	16.0–27.0

SD: Standard deviation; IQR: Interquartile range.

**Table 3:** Summary of the basic parameters of the different models.

Day	Time	R^2^	Adjusted R^2^	Adjusted 10-Fold cv R^2^	RMSE (μg·m^−3^)	10-Fold cv RMSE (μg·m^−3^)
Winter working day	0:00	0.955	0.951	0.947	19.79	29.39
	8:00	0.675	0.644	0.671	43.68	52.64
	18:00	0.829	0.818	0.800	45.48	27.33
Winter non-working day	0:00	0.803	0.777	0.783	44.71	55.42
	8:00	0.732	0.706	0.698	42.75	14.52
	18:00	0.892	0.886	0.880	50.76	32.19
Summer working day	0:00	0.668	0.628	0.592	5.08	9.55
	8:00	0.567	0.510	0.466	8.24	4.84
	18:00	0.354	0.312	0.299	7.72	7.92
Summer non-working day	0:00	0.468	0.434	0.435	5.64	6.51
	8:00	0.635	0.598	0.605	7.87	9.23
	18:00	0.593	0.567	0.574	7.71	6.62

RMSE: Root-mean-square error.

**Table 4:** The LUR models at different times during a winter working day.

Time	Variable	Coefficient *β*	*t*	Sig	VIF	Partial R^2^
0:00	Intercept	−673.896	−15.482	0.000	NA	NA
	Relative humidity	13.277	19.098	0.000	1.683	0.867
	Sports and leisure_2500	0.121	7.653	0.000	1.145	0.073
	Elevation	0.114	3.249	0.003	1.729	0.015
8:00	Intercept	−813.746	−6.303	0.000	NA	NA
	Relative humidity	13.137	7.280	0.000	1.521	0.608
	Primary road_1500	0.001	2.451	0.020	1.334	0.035
	Elevation	0.139	1.750	0.090	1.805	0.032
18:00	Intercept	−384.327	−7.126	0.000	NA	NA
	Relative humidity	13.117	11.583	0.000	2.987	0.549
	Temperature	46.992	7.126	0.000	2.987	0.280

LUR: Land use regression; VIF: Variance inflation factor.

**Table 5:** The LUR models at different times during a winter non-working day.

Time	Variable	Coefficient *β*	*t*	Sig	VIF	Partial R^2^
0:00	Intercept	−531.070	−6.734	0.000	NA	NA
	Relative humidity	10.770	8.755	0.000	1.055	0.574
	Shopping places_2500	1.814	5.122	0.000	4.116	0.143
	Financial banks_3000	−0.122	−2.897	0.007	4.020	0.058
	Woodland_1300	4.649 × 10^−5^	2.058	0.048	1.145	0.028
8:00	Intercept	−303.577	−4.439	0.000	NA	NA
	Relative humidity	6.838	7.460	0.000	1.053	0.620
	Catering services_900	−0.402	−3.131	0.004	1.124	0.055
	Grassland_800	−2.017 × 10^−4^	−2.543	0.016	1.088	0.056
18:00	Intercept	−499.405	−9.598	0.000	NA	NA
	Relative humidity	14.742	15.132	0.000	1.010	0.824
	Shopping places_3000	0.604	4.486	0.000	1.010	0.068

LUR: Land use regression; VIF: Variance inflation factor.

**Table 6:** LUR models at different times during a summer working day.

Time	Variable	Coefficient *β*	*t*	Sig	VIF	Partial R^2^
0:00	Intercept	−130.226	−3.097	0.005	NA	NA
	Residential housing_500	0.296	3.296	0.003	1.230	0.412
	Temperature	6.581	3.860	0.001	1.079	0.193
	Grassland_3000	−3.780 × 10^−6^	−2.179	0.039	1.147	0.063
8:00	Intercept	36.019	14.212	0.000	NA	NA
	Secondary road_300	0.009	4.374	0.000	1.908	0.363
	Financial banks_3000	−0.023	−3.168	0.004	3.397	0.082
	Government agencies_300	0.294	2.099	0.044	2.050	0.065
	Life services_300	1.148	3.706	0.001	1.425	0.058
18:00	Intercept	40.454	20.623	0.000	NA	NA
	Residential housing_300	0.665	2.634	0.013	1.114	0.257
	Scenic spots_3000	0.024	2.157	0.039	1.114	0.097

LUR: Land use regression; VIF: Variance inflation factor.

**Table 7:** LUR models at different times during a summer non-working day.

Time	Variable	Coefficient *β*	*t*	Sig	VIF	Partial R^2^
0:00	Intercept	−32.405	−1.142	0.262	NA	NA
	Residential housing_2500	0.009	2.382	0.023	1.738	0.398
	Temperature	2.553	2.041	0.050	1.738	0.069
8:00	Intercept	−55.293	−1.501	0.144	NA	NA
	Medical care_300	1.154	3.411	0.002	1.330	0.456
	Public facilities_2000	0.036	2.883	0.007	1.185	0.116
	Relative humidity	1.049	2.283	0.030	1.188	0.063
18:00	Intercept	25.358	9.394	0.000	NA	NA
	Science and education places_500	0.188	3.192	0.003	1.710	0.510
	Primary road_2500	5.723 × 10^−5^	2.559	0.015	1.710	0.083

LUR: Land use regression; VIF: Variance inflation factor.

## Supplementary Materials

The following are available online at https://www.mdpi.com/article/10.3390/ijerph18116143/s1, Table S1: PM_2.5_ pollutant concentrations in each district of Beijing at different times, Table S2: The largest correlation coefficients of predictors in different buffer radii.

## References

[1] Zhou M., Wang H., Zeng X., Yin P., Zhu J., Chen W., Li X., Wang L., Wang L., Liu Y. (2019). Mortality, morbidity, and risk factors in China and its provinces, 1990–2017: A systematic analysis for the Global Burden of Disease Study 2017. Lancet.

[2] Steinle S., Reis S., Sabel C.E., Semple S., Twigg M.M., Braban C.F., Leeson S.R., Heal M.R., Harrison D., Lin C. (2015). Personal exposure monitoring of PM_2.5_ in indoor and outdoor microenvironments. Sci. Total Environ..

[3] Adams M.D., Kanaroglou P.S. (2016). Mapping real-time air pollution health risk for environmental management: Combining mobile and stationary air pollution monitoring with neural network models. J. Environ. Manag..

[4] Anenberg S.C., Belova A., Brandt J., Fann N., Greco S., Guttikunda S., Heroux M.-E., Hurley F., Krzyzanowski M., Medina S. (2016). Survey of ambient air pollution health risk assessment tools. Risk Anal..

[5] Huang M., Ivey C., Hu Y., Holmes H.A., Strickland M.J. (2019). Source apportionment of primary and secondary PM_2.5_: Associations with pediatric respiratory disease emergency department visits in the U.S. State of Georgia. Environ. Int..

[6] Sima S., Tajrishy M. (2015). Developing water quality maps of a hyper-saline lake using spatial interpolation methods. Sci. Iran..

[7] Gulia S., Kumar A., Khare M. (2015). Performance evaluation of CALPUFF and AERMOD dispersion models for air quality assessment of an industrial complex. J. Sci. Ind. Res..

[8] Meng X., Fu Q., Ma Z., Chen L., Zou B., Zhang Y., Xue W., Wang J., Wang D., Kan H. (2016). Estimating ground-level PM_10_ in a Chinese city by combining satellite data, meteorological information and a land use regression model. Environ. Pollut..

[9] Xiao Q., Chang H.H., Geng G., Liu Y. (2018). An ensemble machine-learning model to predict historical PM_2.5_ concentrations in China from satellite data. Environ. Sci. Technol..

[10] Feng R., Zheng H.-J., Zhang A.-R., Huang C., Gao H., Ma Y.-C. (2019). Unveiling tropospheric ozone by the traditional atmospheric model and machine learning, and their comparison: A case study in hangzhou, China. Environ. Pollut..

[11] Chen Z.-Y., Zhang R., Zhang T.-H., Ou C.-Q., Guo Y. (2019). A kriging-calibrated machine learning method for estimating daily ground-level NO_2_ in mainland China. Sci. Total Environ..

[12] Jerrett M., Arain A., Kanaroglou P., Beckerman B., Potoglou D., Sahsuvaroglu T., Morrison J., Giovis C. (2005). A review and evaluation of intraurban air pollution exposure models. J. Expo. Sci. Environ. Epidemiol..

[13] Briggs D.J., Collins S., Elliott P., Fischer P., Kingham S., Lebret E., Pryl K., Van Reeuwijk H., Smallbone K., Van Der Veen A. (1997). Mapping urban air pollution using GIS: A regression-based approach. Int. J. Geogr. Inf. Sci..

[14] Morley D.W., Gulliver J. (2018). A land use regression variable generation, modelling and prediction tool for air pollution exposure assessment. Environ. Model. Softw..

[15] Beelen R., Hoek G., Vienneau D., Eeftens M., Dimakopoulou K., Pedeli X., Tsai M.-Y., Künzli N., Schikowski T., Marcon A. (2013). Development of NO_2_ and NO_x_ land use regression models for estimating air pollution exposure in 36 study areas in Europe—The ESCAPE project. Atmos. Environ..

[16] Ma X., Longley I., Gao J., Kachhara A., Salmond J. (2019). A site-optimised multi-scale GIS based land use regression model for simulating local scale patterns in air pollution. Sci. Total Environ..

[17] Yang Z., Freni-Sterrantino A., Fuller G.W., Gulliver J. (2020). Development and transferability of ultrafine particle land use regression models in London. Sci. Total Environ..

[18] Saucy A., Röösli M., Künzli N., Tsai M.-Y., Sieber C., Olaniyan T., Baatjies R., Jeebhay M., Davey M., Flückiger B. (2018). Land use regression modelling of outdoor NO_2_ and PM_2.5_ concentrations in three low income areas in the Western Cape Province, South Africa. Int. J. Environ. Res. Public Health.

[19] Weissert L.F., Salmond J.A., Miskell G., Alavi-Shoshtari M., Williams D.E. (2018). Development of a microscale land use regression model for predicting NO_2_ concentrations at a heavy trafficked suburban area in Auckland, NZ. Sci. Total Environ..

[20] Shi T., Hu Y., Liu M., Li C., Zhang C., Liu C. (2020). Land use regression modelling of PM_2.5_ spatial variations in different seasons in urban areas. Sci. Total Environ..

[21] Wang J., Cohan D.S., Xu H. (2020). Spatiotemporal ozone pollution LUR models: Suitable statistical algorithms and time scales for a megacity scale. Atmos. Environ..

[22] Dong J., Ma R., Cai P., Liu P., Yue H., Zhang X., Xu Q., Li R., Song X. (2021). Effect of sample number and location on accuracy of land use regression model in NO_2_ prediction. Atmos. Environ..

[23] Mortamais M., Pujol J., Martínez-Vilavella G., Fenoll R., Reynes C., Sabatier R., Rivas I., Forns J., Vilor-Tejedor N., Alemany S. (2019). Effects of prenatal exposure to particulate matter air pollution on corpus callosum and behavioral problems in children. Environ. Res..

[24] Liu W., Li X., Chen Z., Zeng G., León T., Liang J., Huang G., Gao Z., Jiao S., He X. (2015). Land use regression models coupled with meteorology to model spatial and temporal variability of NO_2_ and PM_10_ in Changsha, China. Atmos. Environ..

[25] Song W., Jia H., Li Z., Tang D., Wang C. (2019). Detecting urban land-use configuration effects on NO_2_ and NO variations using geographically weighted land use regression. Atmos. Environ..

[26] Nori-Sarma A., Thimmulappa R.K., Venkataramana G.V., Fauzie A.K., Dey S.K., Venkareddy L.K., Berman J.D., Lane K.J., Fong K.C., Warren J.L. (2020). Low-cost NO_2_ monitoring and predictions of urban exposure using universal kriging and land-use regression modelling in Mysore, India. Atmos. Environ..

[27] Chan L.Y., Kwok W.S., Chan C.Y. (2000). Human exposure to respirable suspended particulate and airborne lead in different roadside microenvironments. Chemosphere.

[28] Qiu X., Duan L., Gao J., Wang S., Chai F., Hu J., Zhang J., Yun Y. (2016). Chemical composition and source apportionment of PM_10_ and PM_2.5_ in different functional areas of Lanzhou, China. J. Environ. Sci..

[29] Yuan J., Zheng Y., Xie X. (2012). Discovering regions of different functions in a city using human mobility and POIs. Proceedings of the 18th ACM SIGKDD International Conference on Knowledge Discovery and Data Mining.

[30] Yuan Q., Cong G., Ma Z., Sun A., Thalmann N.M. (2013). Time-aware point-of-interest recommendation. Proceedings of the 36th International ACM SIGIR Conference on Research and Development in Information Retrieval.

[31] Han Y.-J., Kim H.-W., Cho S.-H., Kim P.-R., Kim W.-J. (2015). Metallic elements in PM_2.5_ in different functional areas of Korea: Concentrations and source identification. Atmos. Res..

[32] Gao Y., Guo X., Ji H., Li C., Ding H., Briki M., Tang L., Zhang Y. (2016). Potential threat of heavy metals and PAHs in PM2.5 in different urban functional areas of Beijing. Atmos. Res..

[33] Lu T., Lansing J., Zhang W., Bechle M.J., Hankey S. (2019). Land Use Regression models for 60 volatile organic compounds: Comparing Google Point of Interest (POI) and city permit data. Sci. Total Environ..

[34] Gao J., Peng X., Chen G., Xu J., Shi G.-L., Zhang Y.-C., Feng Y.-C. (2016). Insights into the chemical characterization and sources of PM_2.5_ in Beijing at a 1-h time resolution. Sci. Total Environ..

[35] Liu Y., Zheng M., Yu M., Cai X., Du H., Li J., Zhou T., Yan C., Wang X., Shi Z. (2019). High-time-resolution source apportionment of PM_2.5_ in Beijing with multiple models. Atmos. Chem. Phys..

[36] Wang Y., Bao S., Wang S., Hu Y., Shi X., Wang J., Zhao B., Jiang J., Zheng M., Wu M. (2017). Local and regional contributions to fine particulate matter in Beijing during heavy haze episodes. Sci. Total Environ..

[37] Sun Y., Jiang Q., Wang Z., Fu P., Li J., Yang T., Yin Y. (2014). Investigation of the sources and evolution processes of severe haze pollution in Beijing in January 2013. J. Geophys. Res. Atmos..

[38] van Nunen E., Vermeulen R., Tsai M.-Y., Probst-Hensch N., Ineichen A., Davey M., Imboden M., Ducret-Stich R., Naccarati A., Raffaele D. (2017). Land use regression models for ultrafine particles in six European areas. Environ. Sci. Technol..

[39] Henderson S.B., Beckerman B., Jerrett M., Brauer M. (2007). Application of land use regression to estimate long-term concentrations of traffic-related nitrogen oxides and fine particulate matter. Environ. Sci. Technol..

[40] Wilton D., Szpiro A., Gould T., Larson T. (2010). Improving spatial concentration estimates for nitrogen oxides using a hybrid meteorological dispersion/land use regression model in Los Angeles, CA and Seattle, WA. Sci. Total Environ..

[41] Kingham S., Briggs D., Elliott P., Fischer P., Erik L. (2000). Spatial variations in the concentrations of traffic-related pollutants in indoor and outdoor air in Huddersfield, England. Atmos. Environ..

[42] Reyes J.M., Serre M.L. (2014). An LUR/BME framework to estimate PM_2.5_ explained by on road mobile and stationary sources. Environ. Sci. Technol..

[43] Chalermpong S., Thaithatkul P., Anuchitchanchai O., Sanghatawatana P. (2021). Land use regression modeling for fine particulate matters in Bangkok, Thailand, using time-variant predictors: Effects of seasonal factors, open biomass burning, and traffic-related factors. Atmos. Environ..

[44] Cai J., Ge Y., Li H., Yang C., Liu C., Meng X., Wang W., Niu C., Kan L., Schikowski T. (2020). Application of land use regression to assess exposure and identify potential sources in PM_2.5_, BC, NO_2_ concentrations. Atmos. Environ..

[45] Hoek G., Beelen R., de Hoogh K., Vienneau D., Gulliver J., Fischer P., Briggs D. (2008). A review of land-use regression models to assess spatial variation of outdoor air pollution. Atmos. Environ..

[46] Yang H., Chen J., Wen J., Tian H., Liu X. (2016). Composition and sources of PM_2.5_ around the heating periods of 2013 and 2014 in Beijing: Implications for efficient mitigation measures. Atmos. Environ..

[47] Lv L., Chen Y., Han Y., Cui M., Wei P., Zheng M., Hu J. (2021). High-time-resolution PM_2.5_ source apportionment based on multi-model with organic tracers in Beijing during haze episodes. Sci. Total Environ..

[48] Zhang A., Qi Q., Jiang L., Zhou F., Wang J. (2013). Population exposure to PM_2.5_ in the urban area of Beijing. PLoS ONE.

[49] Mölter A., Lindley S., de Vocht F., Simpson A., Agius R. (2010). Modelling air pollution for epidemiologic research—Part II: Predicting temporal variation through land use regression. Sci. Total Environ..

[50] Naughton O., Donnelly A., Nolan P., Pilla F., Misstear B.D., Broderick B. (2018). A land use regression model for explaining spatial variation in air pollution levels using a wind sector based approach. Sci. Total Environ..

[51] Nordio F., Kloog I., Coull B.A., Chudnovsky A., Grillo P., Bertazzi P.A., Baccarelli A.A., Schwartz J. (2013). Estimating spatio-temporal resolved PM_10_ aerosol mass concentrations using MODIS satellite data and land use regression over Lombardy, Italy. Atmos. Environ..

[52] Abernethy R.C., Allen R.W., McKendry I.G., Brauer M. (2013). A land use regression model for ultrafine particles in Vancouver, Canada. Environ. Sci. Technol..

[53] Zhang D.-C., Liu J.-J., Jia L.-Z., Wang P., Han X. (2019). Speciation of VOCs in the cooking fumes from five edible oils and their corresponding health risk assessments. Atmos. Environ..

[54] Jin W., Zhi G., Zhang Y., Wang L., Guo S., Zhang Y., Xue Z., Zhang X., Du J., Zhang H. (2021). Toward a national emission inventory for the catering industry in China. Sci. Total Environ..

[55] He J., Gong S., Yu Y., Yu L., Wu L., Mao H., Song C., Zhao S., Liu H., Li X. (2017). Air pollution characteristics and their relation to meteorological conditions during 2014–2015 in major Chinese cities. Environ. Pollut..

[56] Statheropoulos M., Vassiliadis N., Pappa A. (1998). Principal component and canonical correlation analysis for examining air pollution and meteorological data. Atmos. Environ..

[57] Jin L., Berman J.D., Warren J.L., Levy J.I., Thurston G., Zhang Y., Xu X., Wang S., Zhang Y., Bell M.L. (2019). A land use regression model of nitrogen dioxide and fine particulate matter in a complex urban core in Lanzhou, China. Environ. Res..

[58] Liu C., Henderson B.H., Wang D., Yang X., Peng Z.-R. (2016). A land use regression application into assessing spatial variation of intra-urban fine particulate matter (PM_2.5_) and nitrogen dioxide (NO_2_) concentrations in City of Shanghai, China. Sci. Total Environ..

[59] Dai Q., Bi X., Liu B., Li L., Ding J., Song W., Bi S., Schulze B.C., Song C., Wu J. (2018). Chemical nature of PM_2.5_ and PM_10_ in Xi’an, China: Insights into primary emissions and secondary particle formation. Environ. Pollut..

[60] Karner A.A., Eisinger D.S., Niemeier D.A. (2010). Near-roadway air quality: Synthesizing the findings from real-world data. Environ. Sci. Technol..

[61] Son Y., Osornio-Vargas Á.R., O’Neill M.S., Hystad P., Texcalac-Sangrador J.L., Ohman-Strickland P., Meng Q., Schwander S. (2018). Land use regression models to assess air pollution exposure in Mexico City using finer spatial and temporal input parameters. Sci. Total Environ..

[62] Wang R., Henderson S.B., Sbihi H., Allen R.W., Brauer M. (2013). Temporal stability of land use regression models for traffic-related air pollution. Atmos. Environ..

