# Supplementary material for: High Temporal Resolution Land Use Regression Models with POI Characteristics of the PM2.5 Distribution in Beijing, China
Source: Int J Environ Res Public Health. 2021 Jun 7;18(11):6143. doi: 10.3390/ijerph18116143 (PMC8201188; doi:10.3390/ijerph18116143)
Supplement: Supplementary file 1 [file ijerph-18-06143-s001.zip › ijerph-1169943-supplementary.pdf]

Table S1. $PM_{2.5}$ pollutant concentrations in each district of Beijing at different times.

District	*N*	Winter Working Day			Winter Non-Working Day			Summer Working Day			Summer Non-Working Day		
		0:00	8:00	18:00	0:00	8:00	18:00	0:00	8:00	18:00	0:00	8:00	18:00
Dongcheng	2	234.5	139.0	246.5	326.5	143.0	428.5	42.0	56.0	40.0	37.5	50.5	27.5
Xicheng	2	229.5	94.0	198.5	295.5	116.5	391.5	44.0	62.5	38.5	38.0	59.0	19.0
Haidian	4	144.0	46.0	92.3	121.8	112.3	223.3	45.8	57.8	41.5	33.5	37.5	29.5
Chaoyang	4	223.0	80.0	111.0	185.5	106.3	324.5	38.0	46.0	36.5	40.0	49.8	20.5
Fengtai	4	225.0	92.8	223.3	285.5	127.0	423.5	39.8	43.5	33.3	31.3	42.3	21.0
Shunyi	1	59.0	28.0	197.0	198.0	84.0	139.0	28.0	31.0	38.0	33.0	33.0	10.0
Tongzhou	2	244.0	283.0	247.0	326.5	256.5	510.0	35.0	65.5	42.5	26.0	45.0	17.0
Daxing	3	261.7	236.3	342.0	348.0	351.7	520.3	35.0	44.7	24.7	22.3	36.3	18.7
Fangshan	2	229.0	93.5	309.0	273.0	144.0	579.5	27.0	40.5	39.0	31.5	30.0	19.5
Mentougou	1	47.0	24.0	12.0	116.0	90.0	199.0	31.0	30.0	38.0	25.0	15.0	24.0
Changping	2	18.0	26.0	22.5	104.0	115.0	127.5	30.5	55.0	47.0	24.5	24.0	41.5
Pinggu	2	51.5	95.0	264.5	171.0	224.5	288.0	27.5	40.0	42.0	26.5	31.5	15.5
Miyun	2	19.0	21.5	131.5	93.5	73.0	107.5	24.0	30.0	45.0	27.5	36.0	16.5
Huairou	1	21.0	18.0	145.0	70.0	83.0	116.0	22.0	31.0	46.0	24.0	27.0	23.0
Yanqing	2	46.0	50.5	11.5	140.5	149.0	116.0	16.0	41.0	43.0	22.5	27.5	25.0

Table S2. The largest correlation coefficients of predictors in different buffer radii.

Predictors	Winter Working Day			Winter Non-Working Day			Summer Working Day			Summer Non-Working Day		
	**0:00**	**8:00**	**18:00**	**0:00**	**8:00**	**18:00**	**0:00**	**8:00**	**18:00**	**0:00**	**8:00**	**18:00**
Arable land	−0.191	0.275	0.410*	0.228	0.353*	0.182	−0.449**	−0.194	−0.433*	−0.378*	−0.510**	−0.564**
Garden land	−0.585**	−0.449**	−0.474**	−0.605**	−0.233	−0.583**	−0.460**	−0.393*	0.436**	−0.546**	−0.495**	−0.409*
Woodland	−0.349*	−0.246	−0.189	−0.344*	−0.255	−0.340*	−0.213	−0.280	0.198	−0.294	−0.443**	−0.324
Grassland	−0.581**	−0.391*	−0.217	−0.474**	−0.384*	−0.429*	−0.448**	−0.355*	0.601**	−0.352*	−0.409*	−0.405*
Commercial service land	0.469**	0.342*	0.338	0.444**	0.159	0.369*	0.608**	0.414*	0.302	0.491**	0.480**	0.630**
Primary road	0.520**	0.350*	0.175	0.435**	0.077	0.428*	0.614**	0.424*	0.527**	0.496**	0.614**	0.681**
Secondary road	0.484**	0.291	0.074	0.386*	−0.314	0.294	0.556**	0.497**	−0.334*	0.545**	0.653**	0.638**
Catering services	0.450**	0.271	0.011	0.373*	−0.381*	0.280	0.550**	0.523**	0.385*	0.566**	0.595**	0.639**
Scenic spots	0.199	0.057	−0.253	0.187	−0.255	−0.054	0.414*	0.286	0.450**	0.566**	0.595**	0.660**
Public facilities	0.455**	0.350*	−0.120	0.347*	0.134	0.223	0.594**	0.351*	0.371*	0.572**	0.593**	0.671**
Companies	0.501**	0.343*	0.062	0.307	−0.076	0.342*	0.667**	0.473**	0.386*	0.602**	0.594**	0.684**
Shopping places	0.518**	0.359*	0.142	0.475**	−0.085	0.382*	0.529**	0.402*	0.417*	0.504**	0.663**	0.622**
Transportation	0.486**	0.344*	0.158	0.396*	−0.115	0.336*	0.667**	0.464**	0.398*	0.551**	0.606**	0.699**
Financial banks	0.421*	0.364*	−0.042	0.336*	−0.163	0.273	0.560**	0.339*	0.364*	0.582**	0.576**	0.606**
Science and education places	0.400*	0.231	−0.029	0.321	−0.139	0.237	0.658**	0.590**	0.464**	0.591**	0.625**	0.773**
Commercial and residential housing	0.460**	0.242	0.018	0.352*	−0.148	0.289	0.649**	0.567**	0.485**	0.592**	0.627**	0.703**
Life services	0.476**	0.333	0.124	0.453**	−0.202	0.341*	0.570**	0.531**	0.324	0.540**	0.600**	0.667**
Sports and leisure	0.380*	0.204	−0.186	0.246	−0.183	0.220	0.543**	0.404*	0.410*	0.559**	0.586**	0.685**
Medical care	0.456**	0.288	0.108	0.451**	−0.233	0.337*	0.620**	0.585**	0.445**	0.543**	0.609**	0.693**
Government agencies	0.408*	0.222	0.065	0.469**	0.022	0.294	0.574**	0.421*	0.383*	0.571**	0.608**	0.629**
Temperature	0.599**	0.101	−0.342*	0.009	0.009	−0.204	0.601**	0.312	−0.308	0.656**	0.141	0.140
Relative humidity	0.931**	0.667**	0.783**	0.805**	0.805**	0.955**	−0.407*	−0.238	0.253	−0.158	0.521**	−0.395*
Air pressure	0.485**	0.770**	0.697**	0.683**	0.683**	0.686**	0.468**	0.146	−0.446**	0.162	0.301	−0.237
Wind speed	−0.559**	−0.238	−0.592**	−0.192	−0.192	−0.201	0.414*	−0.117	0.074	−0.022	0.042	0.308
Population	0.511**	0.299	0.083	0.358*	−0.003	0.331	0.722**	0.516**	−0.034	0.533**	0.596**	0.241
Elevation	−0.573**	−0.714**	−0.704**	−0.599**	−0.523**	−0.616**	−0.505**	−0.222	0.464**	−0.135	−0.204	0.546**
Industry source	0.358*	0.203	0.070	0.291	0.065	0.265	0.256	0.199	−0.358*	0.144	0.232	0.467**

* $p < 0.01$; ** $p < 0.05$. This table only shows the maximum correlation coefficients of the same predictor. Other correlation coefficients with different buffer size are not listed.
